# Health status and public health education for internal older migrants in China: Evidence from a nationally representative survey

**DOI:** 10.3389/fpubh.2022.937361

**Published:** 2022-07-22

**Authors:** Wen Zeng, Cui Wang, Hongbo Chen, Beibei Tong, Dan Li, Ziqiu Zou, Peiyuan Liu, Yuanrong Yao, Shaomei Shang

**Affiliations:** ^1^Nursing School of Peking University Health Science Center, Beijing, China; ^2^Guizhou Provincial People's Hospital, Guiyang, China

**Keywords:** health status, older migrants, public health education, public health services, self-rated health

## Abstract

**Background:**

Self-rated health has been widely used as a useful screening tool to subjectively evaluate individuals' health status. Under the context of the rapid growth of aging, there was a dramatic rapid expansion in internal older migrants in China. Serious concerns on the issues of health status continue to attract quite a lot of attention over the past decades. Public health education is one of the most important health care services and methods to improve individuals' health status. However, most previous studies focus on the utilization of public health services such as visiting to doctors, physical examination, and hospitalization. There was limited evidence on the self-rated health and public health education of older migrants.

**Objectives:**

The study aimed to evaluate self-rated health and the associated determinants in older migrants, as well as to gain a deeper insight into the current status of public health education received by older migrants.

**Methods:**

We derived the data from the National Migrants Population Health and Family Planning Dynamic Monitor Survey 2018, a cross-sectional study, for secondary analysis. Internal migrants aged 60 years old or over were included in the study. Self-rated health was the dependent variable, while sociodemographic characteristics were the independent variable. The univariate and multivariate analyses were performed by Stata 15.1.

**Results:**

A total of 5,589 older migrants were included in the study. Eighty-two percentage of older migrants reported healthy self-rated health. There was a significant association between age, gender, minzu, education level, monthly income, public health education, and self-rated health (*P* < 0.5). However, the proportion of older migrants that received specific public health education was <50%. The most common approach to receiving public health education was through the leaflet, while <20% of older migrants received public health education through public consultation and one-to-one education.

**Conclusions:**

It was necessary to promote the publicity of public health education in older migrants through easy access and diverse approaches in order to effectively spread health-related knowledge to older migrants to satisfy their health needs and maintain their health.

## Introduction

Self-rated health has been widely used as a screening tool in an abundance of studies (1). Self-rated health is used by individuals to subjectively evaluate their own health status, and it was also proved to be a useful indicator of lifestyle-related health status (2). Previous studies indicate that self-rated health might be a predictor of functional ability, adverse health outcomes such as mental disorders (3) and cardiovascular diseases (4), death risk (5) as well as health-related quality of life (6). To the best of our knowledge, research on self-rated health has been studied on pregnant women (7), working population (8), middle-aged and elderly (9), or migrant workers (10, 11). However, evidence on the self-rated health in older migrants in China is quite limited.

Under the context of globalization and pluralistic development, migration becomes a worldwide phenomenon (12). China has become one of the main countries where there is a large number of internal migrants (13) which is considered as a key driver to change the national socioeconomic and demo-graphical characteristics (14). According to a national survey, the total number of internal migrants was 236 million in 2019 in China (15). Notably, under the impact of the rapid growth of aging (16) and traditional Chinese culture of family union (17), there was a dramatic rapid expansion in the elder migrants whose age was 60 and over, and the total number of such a group had raised from 5.03 million in 2000 up to 13.4 million in 2015 (16). Compared to younger migrants, on one hand, older migrants are facing a higher risk of health-related issues such as physiological dysfunction, resistance reduction, and a higher rate of mortality and morbidity, which leads to a much higher demand for health care (17). On the other hand, the aged migrants tend to be less educated than the younger ones, they might experience more barriers to adapting to a new environment and receiving social or medical support (18). Therefore, serious concerns on the issues of health status continue to attract quite a lot of attention over the past decades since the rapid growth of internal elder migrants (18).

A considerable body of literature has focused on the health-related issues in older migrants. Significantly, plenty of studies accentuated public health services utilization in internal migrants (13, 19–29). One of the main findings of those studies provides preliminary evidence that migrants had higher health care needs than native residents (13). In terms of health care services, however, there is a lack of consensus in the utilization of health care services among older migrants since different studies define health care services in different ways or conducted in various target populations. Generally, health care services in most of those studies were related to medical services such as visits to doctors, physical exams, or the establishment of healthcare records. For example, Zheng et al. (13) found that there were no differences in the health care services utilization between rural-to-urban migrants and the urban residents. In their study, health care services were referred to visiting doctors. While in the study conducted by Wen et al. (22), hospitalization represented the health care services, and they found the utilization of health care services was significantly different between female migrants and native residents. Xi et al. (18) referred health care services to medical care such as outpatient care, emergency room admission, inpatient care, preventive care, dental care, and medication use, and the results of their study suggested that there were local-migrant gaps in the utilization of those health care services.

Public health education is one of the most important health care services and methods to improve individuals' health literacy and health status. Interestingly, research on public health education has been quite limited dated. In early 2014, the Chinese government pronounced a National Health Literacy Promotion Action Plan (2014–2020) (30). The National Health Literacy Promotion Action Plan (2014–2020) clearly pointed out that health-related government departments and institutes should take measures with multiple forms to deliver health education related to chronic diseases, infectious diseases, occupational diseases, mental health, reproductive, maternal & child health care, and public emergency in order to improve public's health literacy and their health status. Particularly, the National Health Literacy Promotion Action Plan (2014–2020) states clearly that public health education programs should focus on women, children, the aged, the disabled, and migrants. Despite strong policies recommendation, less is known about the public health education received by older migrants at a national level. Although the research by Zeng and Chen (21) estimated the odds of public health education received by migrants, their study compared the difference of public health education in young generation migrants and older migrants. Another study on health education explored some related determinants of public health education received in migrants (19). Besides, the association between public health education and self-rated health has been overlooked.

Thus, the aim of the current study was to better understand the self-rated health status and health education in internal migrants aged over 60 in the inland of China, also we aimed to explore some determinants related to self-rated health. Based on the results of the current study, we aimed to provide some insights for health policies makers into consideration in order to improve older migrants' access to receive public health education as well as to improve their health status.

The main research questions in the current study were as follows: (1) Do Older migrants have a low level of self-rated health? (2) What were determinants of self-rated health in internal older migrants? (3) Was the access to public health education for older migrants inadequate?

## Materials and methods

### Design, setting and participants

The current study was a secondary analysis. We derived the data from the National Migrants Population Health and Family Planning Dynamic Monitor Survey 2018 which was conducted by the National Health Commission of China in 2018. The survey is a cross-sectional study that used a probability proportionate to size (PPS) sampling method to do such a survey in 31 provinces and the Xinjiang Production and Construction Corps in the inland of China. The target population in the survey was nationally representative and was aged 15 years old and over. In addition, the target population had been living in the current city for at least 1 month or above, but did not get the household registration of the host city. In total, the sample of the National Migrants Population Health and Family Planning Dynamic Monitor Survey 2018 contained 152,000 persons.

The aim of the current study was to estimate the self-rated health status and its' determinants in the older internal migrants. Internal migrants are defined as individuals who separate from the town or street of their household residence for more than half a year, excluding those living in a different town/district in the same city as their household residence. While the older internal migrants refer to internal migrants aged 60 years old or over (16).

### Dependent variables

Self-rated health was selected to be the dependent variable. In the survey, participants were asked “How is your health state.” The answer would be “1 = healthy,” “2 = generally healthy,” “3 = unhealthy but with self-care ability,” “4 = unhealthy without self-care ability.” In our study, we classified self-rated health into a binary variable. In other words, the answer if the participants chose “healthy” or “generally healthy” would be classified as “healthy,” otherwise, it would be classified as “unhealthy.”

### Independent variables

In the current study, sociodemographic characteristics such as age, gender, minzu (ethnicity), education level, marital status, migrating region, monthly income, the establishment of the health record, signing family doctor service contracts, and medical insurance were the independent variables. Public health education was assessed by those questions “Have you ever received public health education related to chronic diseases, infectious diseases, occupational diseases, mental health, reproductive, maternal & child health care, public emergency or other health-related education?” The answer was “yes” or “no.” In the final analysis process, we classified it into “yes” if the participants chose any “yes,” while it would be classified into “no” if all of the answers were “no.”

### Data analysis

We used Stata 15.1 to perform the data analysis. When describing the continuous data, the mean and standard deviation would be used. The categorical variables were demonstrated as frequency and percentages. The Chi-square test was used to assess the difference of distribution in sociodemographic variables between the healthy group and the unhealthy group. At the stage of multi-factor analysis, a generalized linear model, Poisson regression with robust variance estimate, was used to analyze the association between independent variables and self-rated health. The significance level was set at *P* < 0.05.

## Results

### Sociodemographic characteristics of the participants

Totally, there were 5,589 older migrants included in this study. The median age was 64 years old. More than 57% of subjects were male, and the majority ethic of individuals was Han (nearly 92%). Most older migrants were less educated, and more than 75% of the older migrants graduated from middle school or below, and 16.5% from high school, while <8% from junior college or above. Most of the older migrants were married (84.52%). In terms of migrating regions, approximately 45% of older migrants floated cross-province. The median monthly income was 4,500 Yuan (RMB). Only 32.39% of subjects established health records, while more than 50% were without health records. Regarding making family doctor service contracts, <18% of older migrants signed such a primary health care program. Approximately 94% of older migrants had medical insurance. Although most older migrants believed themselves healthy, 18% of whom reported unhealthy.

### Determinants of self-rated health in older migrants

In order to explore the distribution difference of self-rated health in older migrants, we performed a Chi-square test. Demonstrated in Table 1, there was a significantly different distribution in factors including age, gender, minzu, education level, marital status, migrating area, average monthly income, the establishment of the health record, public health education received between healthy group and unhealthy group (*P* < 0.05). However, we did not find a difference of distribution in making family doctor service contracts or having medical insurance between the healthy and unhealthy groups (*P* > 0.05). In order to determine the influencing factors of self-rated health in older migrants, we included all the independent variables in the generalized linear model. Shown in Table 2, the results of generalized linear model indicated that there was a significant association between age, gender, minzu, education level, migrating region, monthly income, public health education, and self-rated health. Specifically, compared with youngest-old (31) migrants whose age was between 60 and 69 years old, the incidence rate ration (IRR) of being unhealthy was 1.56 (95% CI: 1.37, 1.77; *P* < 0.001) in those of middle-old aged from 70 to 79 years old, while the IRR was 2.53 (95% CI: 2.01, 3.18; *P* < 0.001) in those of oldest-old aged 80 years old and over. The IRR was 1.34 (95% CI: 1.20, 1.50; *P* < 0.001) in female older migrants when compared with male ones. Regarding the education level, older migrants who graduated from primary school or below got a higher risk to be unhealthy (IRR = 1.81; 95% CI: 1.30, 2.54; *P* < 0.001) than that from junior college and above. As for monthly income, the IRR of those who had a monthly income of 4,501–7,800 Yuan, 2,501–4,500 Yuan, <2,500 Yuan was, namely, 1.26 (*P* = 0.029), 1.53 (*P* < 0.001), 2.46 (*P* < 0.001) when compared with those of more than 7,801 Yuan. Demonstrated from Table 2, individuals who did not receive public health education got a higher likelihood of being unhealthy (IRR = 1.46, *P* < 0.001) than those who received public health education.

**Table 1:** Distribution difference between healthy and unhealthy group.

	**Unhealthy (%)**	**Healthy (%)**	χ^2^	***P***
**Age (year)**				
60–69	709 (12.69)	3,838 (68.67)	117.2	<0.001
70–79	242 (4.33)	673 (12.04)		
≥80	55 (0.98)	72 (1.29)		
**Gender**				
Male	477 (8.53)	2,728 (48.81)	49.45	<0.001
Female	529 (9.47)	1,855 (33.19)		
**Minzu**				
Han	893 (15.98)	4,245 (75.95)	16.55	<0.001
Minority	113 (2.02)	338 (6.05)		
**Education level**				
Diploma or above	34 (0.61)	373 (6.67)	170.42	<0.001
High school	101 (1.81)	821 (14.69)		
Middle school	226 (4.04)	1,469 (26.28)		
Primary school or below	645 (11.54)	1,920 (34.35)		
**Marriage status**				
Widowed	171 (3.06)	495 (8.86)	32.23	<0.001
Divorced	17 (0.30)	108 (1.93)		
Unmarried	16 (0.29)	58 (1.04)		
Married	802 (14.35)	3,922 (70.17)		
**Migrating region**				
Within city	268 (4.80)	867 (15.51)	47.48	<0.001
Cross-city	375 (6.71)	1,571 (28.11)		
Cross-province	363 (6.49)	2,145 (38.38)		
**Monthly income (Yuan)**				
≥7,801	135 (2.42)	1,253 (22.42)	259.11	<0.001
4,501–7,800	184 (3.29)	1,213 (21.70)		
2,501–4,500	245 (4.38)	1,154 (20.65)		
≤ 2,500	442 (7.91)	963 (17.23)		
**Health document**				
Yes	363 (6.49)	1,447 (25.89)	11.01	0.004
No	534 (9.55)	2,694 (48.20)		
Unclear	109 (1.95)	442 (7.91)		
**Family doctor service contracts**				
Yes	200 (3.58)	783 (14.01)	4.55	0.103
No	711 (12.72)	3,367 (60.24)		
Unclear	95 (1.70)	433 (7.75)		
**Health-related education**				
Yes	653 (11.68)	3,390 (60.65)	33.83	<0.001
No	353 (6.32)	1,193 (21.35)		
**Medical insurance**				
Yes	941 (16.84)	4,310 (77.12)	0.88	0.646
No	61 (1.09)	249 (4.46)		
Unclear	4 (0.07)	24 (0.43)		


**Table 2:** Outcome of generalized linear model.

	**IRR**	**Robust Std. Err.**	**Z**	***P***	**95% CI**
**Age**					
60–69	1.00 (base)				
70–79	1.56	0.10	6.8	<0.001	(1.37, 1.77)
≥80	2.53	0.30	7.89	<0.001	(2.01, 3.18)
**Gender**					
Male	1.00 (base)				
Female	1.34	0.08	5.13	<0.001	(1.20, 1.50)
**Minzu**					
Han	1.00 (base)				
Minority	1.28	0.11	2.9	0.004	(1.08, 1.51)
**Education level**					
Diploma or above	1.00 (base)				
High school	1.17	0.22	0.84	0.403	(0.81, 1.68)
Middle school	1.23	0.22	1.2	0.230	(0.88, 1.74)
Primary school or below	1.81	0.31	3.48	0.001	(1.30, 2.54)
**Marital status**					
Widowed	1.00 (base)				
Divorced	0.81	0.19	−0.87	0.383	(0.51, 1.29)
Unmarried	0.87	0.20	−0.61	0.544	(0.56, 1.36)
Married	1.03	0.08	0.4	0.687	(0.89, 1.20)
**Migrating regions**					
Within city	1.00 (base)				
Cross-city	0.94	0.06	−0.83	0.404	(0.83, 1.08)
Cross-province	0.8	0.06	−3.21	0.001	(0.69, 0.91)
**Monthly income (Yuan)**					
≥7801	1.00 (base)				
4501-7800	1.26	0.13	2.18	0.029	(1.02, 1.55)
2501-4500	1.53	0.16	4.13	<0.001	(1.25, 1.86)
≤ 2500	2.46	0.24	9.32	<0.001	(2.04, 2.97)
**Health records**					
Yes	1.00 (base)				
No	0.9	0.07	−1.39	0.165	(0.78, 1.04)
Unclear	1.0	0.12	0.01	0.991	(0.8, 1.26)
**Family doctor service contracts**					
Yes	1.00 (base)				
No	0.97	0.08	−0.41	0.680	(0.82, 1.14)
Unclear	0.81	0.11	−1.59	0.112	(0.62, 1.05)
**Health-related education**					
Yes	1.00 (base)				
No	1.47	0.09	6.60	<0.001	(1.31, 1.65)
**Medical insurance**					
Yes	1.00 (base)				
No	1.05	0.12	0.43	0.670	(0.84, 1.32)
Unclear	0.75	0.34	−0.62	0.534	(0.31, 1.84)
_cons	0.06	0.01	−13.95	<0.001	(0.04, 0.09)

*IRR, incidence rate ratio; Robust Std. Err: Robust standard error; CI, confidence interval*.

### Access to public health education in older migrants

In total, 72.34% of older migrants received health-related education. However, as presented in Table 3, the rate of receiving specific public health education in older migrants were rather low. As can be seen from Table 3, the proportion of older migrants without receiving public health education related to occupational diseases, infectious diseases, reproductive, maternal & child health care, chronic diseases, public emergency and other health care was, namely, 78.05%, 69.30%, 79.98%, 57.52%, 83.16%, 79.91%, and 82.88%. Table 4 showed the forms of public health education delivered in older migrants. The most common form was leaflet (43.42%), which followed by public science gallery (37.39%), health lecture (33.58%), public consultation (17.80%), text message (14.13%), while only 9.86% of older migrants received one-to-one health education.

**Table 3:** Public health education received in older migrants.

	**Yes**	**No**
Occupational diseases	1,227 (21.95%)	4,362 (78.05%)
Infectious diseases	1,716 (30.70%)	3,873 (69.30%)
Reproductive, maternal & child health care	1,119 (20.02%)	4,470 (79.98%)
Chronic diseases	2,374 (42.48%)	3,215 (57.52%)
Mental health	941 (16.84 %)	4,648 (83.16%)
Public emergency	1,123 (20.09%)	4,466 (79.91%)
Other health care	957 (17.12%)	4,632 (82.88 %)

**Table 4:** Approaches of public health education delivered in older migrants.

	**Frequency**	**Percentage (%)**
Health lectures	1,877	33.58
Leaflets	2,427	43.42
Public science galleries	2,090	37.39
Public consultation	995	17.8
Text messages	790	14.13
One-to-one education	551	9.86
Other forms	912	16.32

## Discussion

In the current study, we estimated the self-rated health status of 5,589 older migrants. Interestingly, we found that 82% of the older migrants in the current study felt they were healthy, which was higher than we had expected. However, such a finding was similar to that in the study of Wei et al. (32). We also found that there was a significant relationship between age, gender, minzu, education level, monthly income, public health education and self-rated health in older migrants.

To be specific, older migrants with older age got a higher relative risk to be unhealthy. Our results provided additional support to the conclusion that age predicted self-rated health of older migrants (33). With the increase of age, individuals' physical function declines gradually which leads to higher prevalence rates of the ability of daily living disability, other daily function disorders, or mortality (34). As a result, the oldest older migrants had more likelihood of having poor self-rated health.

Unsurprisingly, in the present study, monthly income was observed as one of the predictors of self-rated health in older migrants. In other words, a significant positive association between monthly income and self-rated health was detected. More specifically, older migrants with lower income had a higher likelihood to report poor self-rated health, which was in line with findings in previous research (35). One main possible reason would be that people with a higher monthly income had more sufficient abilities and resources to live healthier lifestyles, to take an active part in screening programs, and deal with unexpected medical events (35). Thus, older migrants with higher economic status were more likely to report good self-rated health.

There was an ongoing debate on the relationship between gender and self-rated health. Li et al. (36) pointed out that male migrants were more likely to report poor self-rated health. However, our study showed that females had a higher risk of bad self-rated health. We supposed that the target subjects were different which might lead to such a difference. In the study of Li et al. (36), the age of the participants was from 19 to 65 years old, while that of our study was over 60 years old. In terms of gender difference in self-rated health, female older migrants in our study had lower monthly income (mean: 6,284.18) than male ones (mean: 6,384.95), which might impact their self-rated health. To some degree, such a difference confirmed our finding that monthly income predicted self-rated health of older migrants. Another possible reason might be that female older migrants might be much more sensitive and have less tolerance to suffering such as pain or other disordered symptoms (37). Thus, female older migrants might report poor self-rated health.

Additionally, minzu was detected as a determinant of self-rated health in internal older migrants. Compared with those of Han ethnicity, older migrants with ethnic minorities had a higher risk to report unhealthy. To our best knowledge, in China, there was a big gap of inequalities in the social economy, access to health care resources, employment status, and social connections between ethnic minorities and Han ethnicity (38). Such health inequalities might lead to poor health outcomes in ethnic minority migrants.

Notably, as mentioned above, our findings revealed that there was a significant positive relationship between public health education and self-rated health. Compared to older migrants who received public health education, those without receiving any public health education got a higher likelihood of reporting unhealthy. Empirical studies suggest that the elderly have a higher demand for health-related services with the increase of age. Additionally, their demand for such services tends to be urgent and diverse to maintain their health (33). Public health education, as one of the most basic public health services, is believed to be an effective and fair way to improve individuals' health knowledge and health literacy, which leads to improving their health behaviors to maintain health status (33, 38). Overall, we found that the proportion of older migrants who received public health education was as high as 72.34%, which was a little higher than the findings in the research conducted by Wang et al. (39). We defined the public health education receiving as receiving any kind of public health education, and we suppose such a broad definition led to a high proportion of public health education receiving detected. However, when analyzing the proportion of public health education related to a specific kind of disease, the finding was not as positive as the overall result. To be specific, <50% of older migrants in the study received public health education related to chronic diseases, although it made the highest rate among all kinds of public health education received by older migrants in our study. What's worse, only 16.84% of older migrants received public health education about mental health. To some degree, the finding of our study reflected that the availability and access to public health education in older migrants was at a rather low level. Notably, the Chinese government has stressed building a national healthy society since 2016. According to Healthy China 2030, public health is placed in a national strategic position and it is listed as a precondition of economic and social development (40). To be dated, there was a considerable body of studies stressing the importance of policies to improve the access to public health services for migrants. Unfortunately, those policies mainly focused on younger migrant workers and their children, while the older migrants are largely left behind by those policies to protect the rights and benefits to enjoy those public health services (16, 24). As a result, the public health education services could not finally expand for the whole population.

As for the approach of receiving public health education, leaflet was the most common way. 43.42% of older migrants in the survey received public health education through the leaflet, followed by public science gallery, health lecture. Surprisingly, the proportion of older migrants who received public health education through public consultation and one-to-one education was only 17.8 and 9.86%. On one hand, our results indicated that public health education did not get much publicity. On the other hand, public health education providers did not clearly take older migrants' preferences and abilities to receive health-related knowledge. In our study, over 80% of older migrants were with the education of middle school or below. Compared to younger migrants, older migrants had a lower education background. Therefore, it might be much easier to receive health knowledge through traditional educational methods such as health lectures, public consultation, or one-to-one education rather than through leaflets or public science galleries that required older migrants with higher self-learning abilities (41).

Under the context of the rapid increase of the older internal migrants, it highly deserves to continue to attract attention to services and management capacity for such a huge group (16). Strategies on public health care education should be emphasized in order to ensure older migrants could easily be accessed to health-related services. Based on the findings of our study, we suggest that government and public health education providers should clearly take older migrants' sociodemographic characteristics and their abilities into consideration so that they could easily receive such kinds of knowledge. Especially, primary care providers, who play a core role in health maintenance and promotion in communities, are responsible for the effective delivery of public health education. It is necessary and urgent to develop and provide public health education programs with lower costs and more diverse ways (41) in order to improve older migrants' health status.

### Limitations and recommendations for future studies

One of the main limitations in our study was that we could only gain an insight into the health status at a precise moment, but could not get a deeper insight into that over time due to the nature of the cross-sectional design. Another limitation was that the current study derived data from a national survey for a secondary analysis, which prevented us from exploring the interlinkages between factors such as life events, life course trajectories, cross-cultural adjustment, social integration, social support and self-rated health in older migrants. We suggest that future research could do further explore with more variables in order to get better understand the determinants of self-rated health, which would help policymakers and primary health care providers to develop more precise and easier accepted health strategies to help older migrants realize healthy and positive aging.

## Conclusions

The current study estimated the self-rated health status and explored some possible influencing factors in older migrants. In conclusion, most older migrants reported good self-rated health. Factors such as age, gender, monthly income, minority, and public health education predicted self-rated health in older migrants. Despite the positive results on self-rated health, the proportion of public health education related to a specific kind of disease was rather small. Moreover, the approaches of receiving public health education seemed to be less easy and diverse. Based on the findings of our study, we suggest that it is necessary for policymakers and primary health care providers to develop and deliver more precise and easier accepted public health education programs to help older migrants realize healthy and positive aging.

## Data availability statement

Publicly available datasets were analyzed in this study. This data can be found here: https://www.chinaldrk.org.cn/wjw/#/home.

## Ethics statement

Ethical review and approval was not required for the study on human participants in accordance with the local legislation and institutional requirements. Written informed consent for participation was not required for this study in accordance with the national legislation and the institutional requirements.

## Author contributions

WZ, SS, and YY conceived and designed the study. CW, HC, and DL collected, input and checked the data. BT, ZZ, and PL analyzed the data. WZ drafted the manuscript. All authors read and approved the final manuscript. All authors contributed to the article and approved the submitted version.

## Funding

This study was funded by the Ministry of Science and Technology of the People's Republic China (2020YFC2008801 and 2020YFC2008800), National Natural Science Foundation of China, Guizhou (81860245), and Science and Technology Platform and Talent Team Planning Project ([2019]5664), which offered support in the process of study design and financial support for publication.

## Conflict of interest

The authors declare that the research was conducted in the absence of any commercial or financial relationships that could be construed as a potential conflict of interest.

## Publisher's note

All claims expressed in this article are solely those of the authors and do not necessarily represent those of their affiliated organizations, or those of the publisher, the editors and the reviewers. Any product that may be evaluated in this article, or claim that may be made by its manufacturer, is not guaranteed or endorsed by the publisher.
